# Patient-centered assessment of treatment for alpha-1 antitrypsin deficiency: literature review to identify concepts and measures for people with alpha1-antitrypsin deficiency

**DOI:** 10.1186/s13023-025-03592-9

**Published:** 2025-02-22

**Authors:** Ekin Seçinti, Karolina Schantz, Laure Delbecque, John Krege, Rikki Mangrum, Sarah E. Curtis

**Affiliations:** 1 Eli Lilly and Company, Indianapolis, USA; 2 Vector Psychometric Group, LLC, Chicago, USA; 3 Vector Psychometric Group, LLC, Chapel Hill, NC USA

**Keywords:** Patient-reported outcome, Measurement, Literature review, Alpha-1 antitrypsin deficiency

## Abstract

**Background:**

Alpha-1 antitrypsin deficiency (AATD) is a genetic disorder that can result in a range of illnesses, with chronic obstructive pulmonary disease (COPD) being one of the most common. Although some people obtain genetic testing that identifies AATD, many people are unaware that they have AATD until they develop COPD, often at a younger age than is typical. Treatment for AATD consists primarily of augmentation with AAT, requiring weekly infusions of blood products for most patients. This treatment can slow disease progression and improve symptoms, but is burdensome; thus, people with AATD could benefit from additional or alternate treatments. However, to guide the development of new treatments, researchers need to identify which outcomes matter to people with AATD.

**Methods:**

We conducted a scoping literature review to better understand patient experiences with AATD and its treatment and identify patient-reported outcome measures (PROMs) used to assess symptoms and impacts in studies of people with AATD.

**Results:**

The review identified 44 concepts related to symptoms and disease burden, grouped into six domains (symptoms, physical function, cognitive function, emotional function, psychosocial function, and treatment burden) and 24 PROMs that have been used in research on AATD. None of the identified measures were developed specifically for people with AATD. Research on patient-focused outcomes was limited, suggesting a significant gap in knowledge.

**Conclusions:**

People with AATD experience a variety of disease-related burdens, but this study showed there is a lack of published, in-depth studies to support selection and evaluation of patient-centered outcomes among populations of people with AATD. A limited number of PROMs have been used in research on AATD or in clinical trials of treatment, including COPD-specific measures that assess symptoms and quality of life and measures of mood, sleep, and general physical and psychosocial functioning. The current study documented the available evidence and compiled a list of potential concepts of interest, but further qualitative and quantitative studies will be needed to understand the outcomes that matter to people with AATD and to evaluate the alignment between these outcomes and available measures.

**Supplementary Information:**

The online version contains supplementary material available at 10.1186/s13023-025-03592-9.

## Background

Alpha-1-antitrypsin (AAT) deficiency (AATD) is a genetic disorder caused by mutations of the SERPINA1 gene. These mutations result in low serum levels of AAT, which is a protease inhibitor that protects tissue from damage by neutrophil proteases [1, 2]. AATD is associated with increased risk of developing lung disease and some genetic variants are associated with more severe disease and higher risk of mortality [3]. AATD also imposes a significant disease and economic burden on patients [4]. People with AATD have a higher risk of lung disease development at a younger age than is typical, especially when compounded by other factors, such as smoking or exposure to airborne pollutants [3]. Although the precise prevalence is challenging to determine, some studies suggest that AATD may be significantly underdiagnosed, or diagnosis may be delayed, due to limited genetic screening practices and inconsistent testing of people with illnesses associated with AATD [1, 5]. Early detection of AATD is vital because therapy to augment AAT and behavioral modifications (e.g., smoking cessation) may provide protection against disease [6].

To date, clinical research on AATD with or without lung disease has focused on epidemiological studies to determine the prevalence of different genotypes, assess specific morbidity and mortality outcomes, and clinical trials of augmentation therapy intended to understand specific aspects of treatment, such as the optimal dosing to prevent lung function deterioration [4, 7]. Although these studies help describe the incidence of AATD within various populations around the world and increase knowledge of AATD as a contributor to lung disease, they provide limited insight into the specific outcomes that matter to patients when considering treatment options. Recent published trials of treatment for AATD have typically included clinical assessments of AAT serum levels and lung function [8–11], with only a few including patient-reported outcome measures (PROMs) such as general assessments of health-related quality of life (HRQOL) or measures of the symptoms and impacts of chronic obstructive pulmonary disease (COPD) [12]. PROMs have been used more often in observational studies that seek to correlate AATD and its varied genotypes to different levels of patient burden from disease, including increased burden that may result from delayed diagnosis or early onset of lung symptoms [13–15].

To better understand patient experiences with AATD and its treatment, this scoping review [16] study aimed to assess two types of published literature on AATD: qualitative research studies or other resources containing testimony from people who have AATD (with or without a diagnosis of lung disease) and observational or clinical research studies of people with AATD that included the use of a PROM. The review aimed to extract data about the health-related concepts that may be relevant to patients when considering treatment options and to identify the PROMs that have been used to evaluate health-related quality of life and functioning outcomes for the AATD population.

## Methods

**Fig. 1:**
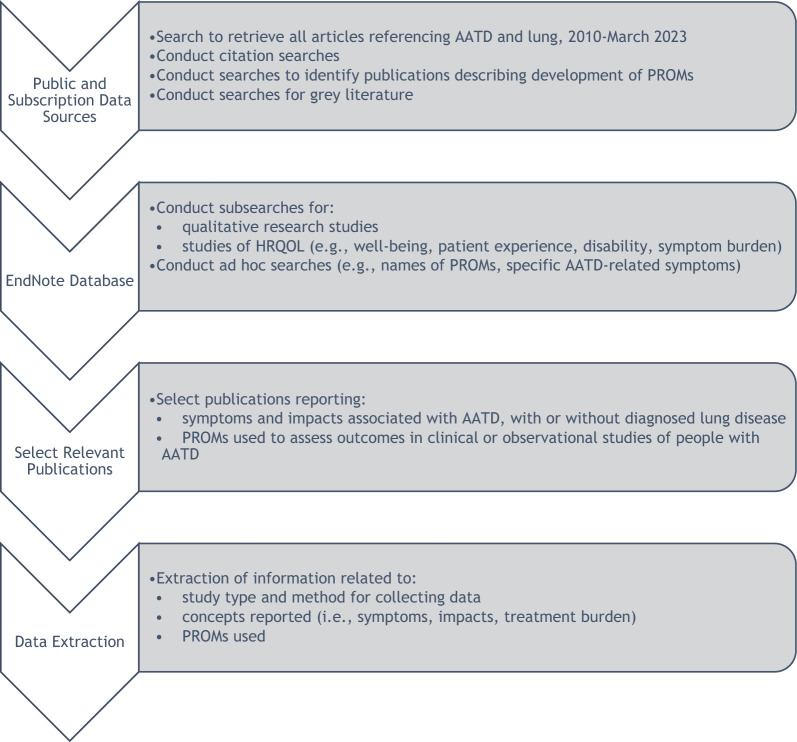
Overview of study steps

A broad scoping strategy for retrieving potentially useful publications was developed because AATD is an uncommon health condition and research on patient-centered outcomes appeared to be limited (Fig. 1. Overview of Study Steps. Searches were conducted in Embase and PubMed, two comprehensive bibliographic databases of health science literature, on February 9, 2023 and March 13, 2023 respectively, for all publications referencing AATD and lung symptoms or diseases (search strategy provided in Additional file 1, Table 1). All retrieved records were downloaded and combined into a single bibliographic database using EndNote 20 software. This procedure resulted in a study-specific EndNote database of English language publications dating from 2010 or later that were indexed by Embase or PubMed and included at least one term for AATD and one term for lung function, disease, or impairment. This EndNote database was then used to execute a series of searches to identify publications on specific topics of interest (search strategies are provided in Additional file 1, Table 2). These searches were intended to retrieve articles that addressed one or more of the following: qualitative research studies with an AATD population and studies of any type that addressed HRQOL, such as patient experiences, quality of life, functioning or disability, or symptom burden, in people with AATD. Bibliographic records that were responsive to these search strategies were grouped and reviewed for adherence to several inclusion and exclusion criteria, shown in Table 1. Finally, to identify articles about PROMs that have been used in AATD populations, the EndNote database was searched for the names and acronyms of PROMs as they were identified during the reviews of titles and abstracts and during full-text review.

**Table 1:** Inclusion and exclusion criteria for qualitative and HRQOL studies

Include	Exclude
Patient population include adults (i.e., age ≥ 18 years)	Population includes children and/or adolescents (i.e., age < 18 years)
Report a study of people who have AATD and lung disease that includes qualitative appraisal of patient experiences with the condition or its treatment	Editorials, clinical practice guidelines, commentary
Report a clinical or observational study focused on assessing HRQOL in people with AATD (with or without a lung-related diagnosis) using a self-reported outcome measure	Human cell or genetic studies, population prevalence studies with no reporting of symptoms or impacts of AATD, single case studies, or other studies not relevant to the review aims (i.e., do not include substantive information about patient-centered outcomes)
	Studies not specifically focused on patients with AATD or focused only on patients with AATD and other disease exacerbations (e.g., liver or cardiac disease)
	Systematic reviews of objective outcomes, such as blood tests or tests of lung capacity

Despite the size of the EndNote database, the topical searches produced limited useful results. Consequently, a series of additional searches and supplementary strategies were used to identify additional publications relevant to understand people’s experiences with AATD. These ad hoc searches included searches for new terms identified during article review (e.g., the names of specific symptoms or PROMs); ‘snowball’ searches for articles identified in references lists; searches using Google and Bing search engines; use of the ‘similar article’ function in PubMed; and using Google Scholar and Web of Science to retrieve and review lists of articles that cited publications that had been identified as highly relevant during article review. All primary and ad hoc searches were conducted by a trained and experienced research librarian and items retrieved by added searches were added to the EndNote database. Finally, to further improve the scope of the review due to the limited retrieval of useful publications, the inclusion criteria were adjusted to permit the inclusion of two key source documents that do not qualify as research studies: the US Food and Drug Administration’s (FDA) *Voice of the Patient* report [17], which reports on a public meeting held in September, 2015, to hear from patients with AATD, and the *Big Fat Reference Guide* [18], a patient-centered resource guide developed by AlphaNet, the advocacy affiliate of the Alpha-1 Foundation.

Two analysts independently reviewed all publications to determine whether they reported information about AATD-related patient experiences, including experiences of symptoms or impacts of lung impairment/disease among people with AATD or the use of PROMs to assess symptoms and impacts in an AATD patient population. The two analysts then extracted data from relevant publications. Extracted variables included aspects of the source material (e.g., study type), terms or phrases used to describe symptoms or impacts of AATD or AATD-related lung disease, and information about any PROMs used to assess symptoms or impacts.

To develop a broad conceptual understanding of patient experiences with AATD, specific terms and phrases used in publications to describe symptoms and impacts of AATD or AATD-related lung disease were classified to group similar items together with a unified concept label. For example, the terms ‘improved sleep,’ ‘sleep disturbance,’ and ‘sleep quality’ were grouped together under the overarching concept label ‘sleep.’ The overarching concepts were subsequently grouped together into domains based on their similarity and interrelationships. For example, concepts like ‘cough’ and ‘dizziness’ were placed together in the Symptom domain, while concepts like ‘sleep’ and ‘basic mobility’ were grouped under the Physical Function domain.

To develop a more detailed understanding of the PROMs that have been used with the AATD population in research studies, details about measures (e.g., number of items, recall period, scoring) were extracted from publications when available. Gaps in information were addressed by reviewing original publications describing the instrument or available information provided by measure management organizations (e.g., Mapi Research Trust). For each measure identified, a supplementary search of ClinicalTrials.gov, a central repository of clinical trial protocol records, was conducted to determine whether the PROM had been used in any clinical trials of treatment for AATD that began 2010 to March 2023; trials that had been suspended or withdrawn were excluded.

## Results

**Fig. 2:**
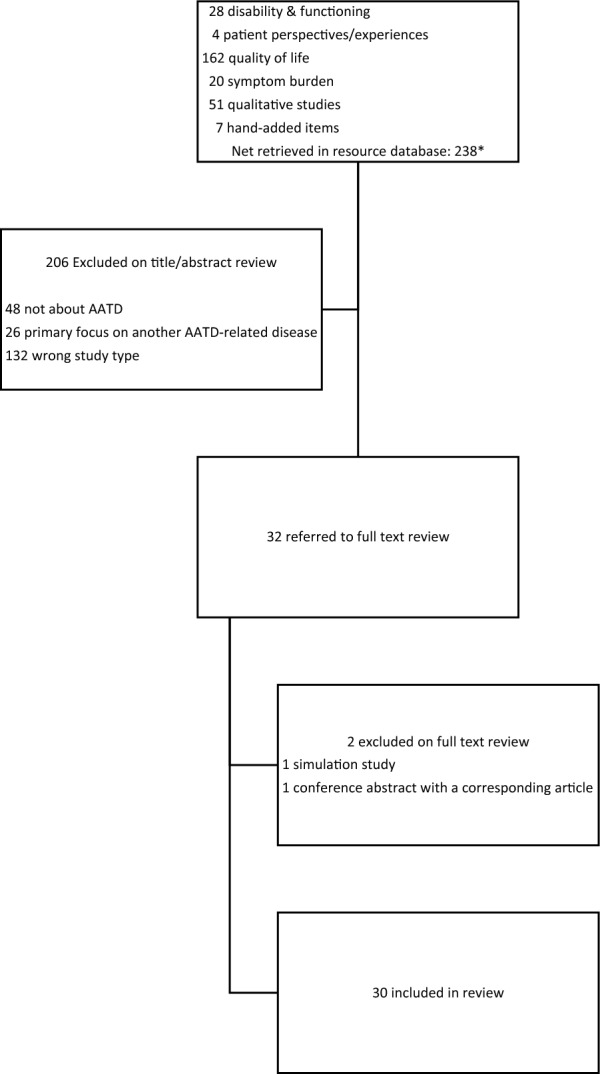
PRISMA diagram for qualitative and HRQOL review. *Sums to more than 238 because some items were retrieved by more than one search strategy

The baseline searches retrieved 2572 non-duplicative publication records from Embase and PubMed that were added to EndNote. EndNote’s automated algorithms identified one publication that had been retracted and 21 publications that were duplicates, resulting in 2550 publications. Seven additional publications relevant to the aim of understanding AATD patient experiences were identified through ad hoc searches, resulting in a total of 2557 publications in the EndNote database. Search results described below were obtained by conducting subsequent searches within this EndNote database. As shown in the preferred reporting items for systematic reviews and meta-analyses (PRISMA) diagram (Fig. 2), searches for qualitative studies and studies reporting on symptom burden, patient experiences, quality of life, and disability or functioning, plus publications retrieved by added searches, produced a total of 238 publications from the EndNote database. On review of titles and abstracts, 206 publications were excluded and 32 were included in the full text review. Two publications were excluded during full text review, resulting in a set of 30 publications included in data extraction [17–46]. These 30 publications included reviews (n = 7), observational studies (n = 14), longitudinal studies (n = 3), qualitative studies (n = 4), and report/guides containing patient testimony (n = 2).

### Concepts in AATD patient experience

A total of 112 descriptive terms for patient-centered health experiences related to symptoms and disease burdens were found across the publications reviewed (Additional file, Table 3). Conceptually similar or highly related terms were grouped together. For example, Wienke et al. refer both to behavior modification related to susceptibility to disease and to avoidance behaviors regarding environmental exposure [31], which were grouped together with other similar terms under the concept label ‘behavior burden and vigilance.’ After grouping similar terms together, a total of 44 uniform concepts were identified and grouped into 6 domains, including four domains for functioning, one for symptoms, and one for treatment burden (Table 2).

**Table 2:** Domains and concepts identified from the literature

Domain	Concept(s)	Examples
Symptoms	Congestion	Feelings of lung congestion
	Cough	Frequency and severity of cough or lung spasms
	Cyanosis	Cyanosis due to low oxygen
	Dizziness	Experiences of dizziness, likely due to low oxygen
	Dyspnea	Shortness of breath, including reduced respiratory rate, tightness in the chest, and increased shortness of breath during certain activities
	Edema	Fluid retention or swelling in the extremities
	Exacerbations	Exacerbations of lung disease, which includes transient exacerbations, such as increased cough following brief exposures, as well as serious exacerbations, such as lung infections
	Fatigue/Level of energy^a^	Decreased energy or increased fatigue that results from lung disease due to shortness of breath; lack of stamina; need to rest during activity; experience of fatigue as an initial symptom, before diagnosis with a specific condition
	Headache	Headache, likely caused by low oxygen
	Mental acuity	Cognitive changes or feelings of confusion, likely caused by low oxygen
	Muscle weakness	Muscle weakness, likely caused by low oxygen
	Sputum/Phlegm	Frequency of phlegm production, as well as quantity and color
	Weight loss	Unintentional weight loss
	Wheezing	Wheezing all the time or with infections such as colds
Cognitive function	Ability to concentrate	Ability to concentrate or focus on what you are doing
	Memory	Ability to remember, forgetfulness
	Speech	Slurred speech
Emotional function	Anger^a^	Feelings of anger or frustration about having AATD, lung symptoms, or impairment of functioning
	Anxiety/stress^a^	Experiences of anxiety or stress about the future due to genetic diagnosis; anxiety or stress about developing disease or about disease symptoms; feelings of worry; panic attacks; anxiety about math ability/comprehension (numeracy) related to understanding probabilities associated with a genetic illness
	Depression^a^	Feelings of sadness or depression; feeling fatalistic
	Fear^a^	Feelings of fear related to genetic diagnosis or lung disease; fear for the health of children or future generations
	Guilt^a^	Feelings of guilt associated with having passed a genetic condition on to others
	Irritability	Feelings of irritability associated with symptoms or impairments of functioning
	Mood	Altered mood or mood swings associated with lung symptoms or impairments of functioning
	Sense of control^a^	Ability to feel independent or in control of one’s life; sense of uncertainty or unpredictability associated with AATD or with lung disease symptoms and impacts; the desire for peace of mind or the ability to feel hopeful
Physical function	Ability to participate in regular daily activities	Ability to carry out routine daily activities, such as household activities, errands, etc
	Basic mobility	Ability to walk; ability to carry out activities of daily living; needing assistive devices (e.g., a walker) or help from a caregiver to get around; ability to travel
	Exercise^a^	Ability to exercise after developing lung disease; importance of exercise for limiting disease risk among healthy individuals with AATD
	Exertion	Ability to engage in activities that require exertion (e.g., climbing stairs, walking up a hill)
	Nutrition^a^	Ability to maintain good nutritional status or avoid malnutrition after developing lung disease; importance of nutrition for limiting disease risk among healthy individuals with AATD
	Sleep	Experiences of sleep disturbance or poor sleep quality due to symptoms; daytime sleepiness; desire for better sleep quality
Psychosocial function	Ability to participate in social roles^a^	Ability to carry out family or social roles; difficulty caring for children or pets; ability to work or to work in one’s chosen occupations; impairments of ability to work, including missed workdays due to symptoms or need for medical care
	Behavior burden and vigilance^a^	Engaging in behavior modifications or vigilance related to susceptibility to disease, external risk factors, or exacerbations (e.g., avoiding or ceasing smoking, avoiding airborne particulates); making changes in lifestyle or occupation due to AATD or AATD-related illness
	Impact on other people^a^	Impacts on family members or family relationships that result from sharing a genetic diagnosis with AATD; impacts on family members or other caregivers that result from lung impairment or disease
	Reproductive health^a^	Family planning or pregnancy decisions related to having a genetic diagnosis
	Sexual function	Impacts on libido and sexual functioning that result from AATD-related lung disease
	Social impact^a^	Impacts on social roles and relationships, including strain on relationships following the sharing of a genetic diagnosis
Treatment burden	Cost^a^	Costs of treatment for AATD or COPD
	Physical comfort and convenience of treatment^a^	Comfort and convenience of treatment, including augmentation therapy, supplemental oxygen devices, or inhaled medication; comfort using devices that are worn at night; perspectives on the duration of treatment effect
	Satisfaction^a^	Satisfaction with treatment
	Treatment side effect^a^	Side effects such as treatment reactions or nasal/mouth dryness resulting from supplemental oxygen use
	Treatment time/effort^a^	Travel and therapeutic time required for treatment; need to schedule one’s life around treatments
	Treatment use	Use of acute or ‘rescue’ medications, airway clearance devices or methods, inhaled medications, or supplemental oxygen
	Embarrassment	Embarrassment experienced because of visible equipment, such as an oxygen delivery device

^a^Also occurs amongst people who have AATD but no lung disease

The concepts and domains in Table 2 encompass the experiences of both individuals with AATD and lung disease and those who know they have AATD but do not yet have any lung disease symptoms. The findings suggest that people who know they have AATD but are asymptomatic (generally healthy) may experience emotional impacts (e.g., anxiety, depression) and burdens from living with uncertainty, which may include changes in lifestyle, behavior, and occupation to minimize environmental exposures. People with AATD who have developed lung symptoms experience additional burdens due to the specific symptoms, exacerbations, and impacts of disease, as well as the burden of augmentation therapy to treat AATD.

The symptoms domain includes symptoms of lung disease, some of which may be experienced differently or at an earlier age by individuals with AATD. Sandhaus et al. [36] observed that while exacerbation frequency is similar among people with AATD and COPD and people with COPD who do not have AATD, people with AATD may experience more severe exacerbations, and progression of lung disease in people with AATD is strongly affected by exacerbation frequency. Accordingly, the treatment burdens domain encompasses both burdens of augmentation therapy for AATD and treatments for lung disease.

The cognitive function domain includes the ability to concentrate, memory, and slurred speech, which are symptoms that can occur during acute pulmonary exacerbations in people with AATD and lung disease [18]. The emotional function domain includes multiple concepts that are relevant to both people with lung disease and those who have AATD but are still generally healthy. Anger, anxiety, depression, fear, and loss of control can result from the uncertainty of living with the knowledge that one has AATD and worries about the future impacts of AATD-related illness on oneself and one’s family [41]. Anger, depression, anxiety, stress, and fear are also related to the burden of dealing with lung disease symptoms and impacts on functioning [18, 41]. Irritability and mood changes are mentioned in the AlphaNet guide [18] specifically as potential signs of an acute exacerbation.

The physical function domain includes the impact of lung disease on basic daily activities, activities that require physical exertion and mobility. This domain also includes exercise and nutrition considerations for both those with and without lung disease. Exercise and nutrition are mentioned in the AlphaNet guide [18] for healthy people with AATD as they can help in limiting other health problems that could further exacerbate future lung disease. Difficulty exercising and maintaining adequate nutrition are also mentioned in the AlphaNet guide as limitations for those living with lung disease. Finally, the sleep concept includes general sleep disturbance and sleep apnea, which can be magnified by lung disease.

Many of the concepts in the psychosocial function domain encompass experiences of people with lung disease and healthy people with AATD. Lifestyle adaptations, such as changing jobs or avoiding environmental exposures, required to minimize disease risk or manage lung disease create a burden for people with AATD and can impact their social and family life [19, 20, 47]. Reproductive health or family planning is also a concern for people with AATD due to the fear of passing the gene on to one’s children [17, 31]. Sexual function can be affected by symptoms of lung disease and is discussed in the AlphaNet guide section on managing lung disease [18].

### Patient reported outcome measures identified

The review identified 24 PROMs that have been used in studies of people with AATD and lung disease (Table 3). However, none of the identified measures were developed specifically for people with AATD and the majority have been used infrequently in this population (i.e., 14 measures were referenced in only 1–2 publications). Two PROMs, the St. George’s Respiratory Questionnaire (SGRQ) and the EQ-5D-5L, have also been used in recent clinical trials of AATD treatment (see Table 4). The three most commonly used measures were the COPD Assessment Test (CAT), Modified Medical Research Council dyspnea scale, and the SGRQ. An FDA guidance document [48] specific to the SGRQ was identified during follow-up searches to retrieve information about the PROM’s content and recall period. This document notes that FDA has determined that the SGRQ and the alternate SGRQ-C version (a shorter version tailored specifically for COPD) are suitable for use as a coprimary or secondary endpoint for treatment efficacy assessment in a clinical trial for COPD. The document shares specific recommendations and limitations that pertain to the use of the SGRQ or SGRQ-C in the context of an FDA regulatory review, but does not specifically address suitability for trials within an AATD population. However, the SGRQ has been used for secondary or exploratory endpoints in five recent clinical trials of treatment for people with AATD and lung disease (Table 4).

**Table 3:** Measures identified in the review

Measure name and citation	COA type	Target population	Domains/constructs	No. of items	Response scale	Scoring	Recall period	In AATD literature^a^
Beck Anxiety Inventory [51]	PROM	Adolescents and adults	Cognitive anxiety, somatic anxiety	21	0–3 Likert-type for bothersomeness	Raw score. Minimal anxiety (0–7), mild (8–15), moderate (16–25), and severe (30–63)	1 month	1 publication [41]
Berlin Questionnaire [52]	Diagnostic risk factors, self-reported	People at risk for sleep apnea	Sleep apnea symptoms, sleep apnea impacts, vital statistics	10	Varies by question	Domain scores; patients are at high risk for sleep apnea if two domain scores are positive	None specified	1 publication [53]
Borg Scale [54] (also variously referred to as the Borg CR10 Scale, Borg Dyspnea Scale, Borg Fatigue Scale, and Borg Rating of Perceived Exertion)	Ecological momentary assessment, interviewer-led or PROM	Any; typically used in conjunction with a walk test	Level of effort/exertion, breathlessness, or fatigue while executing physically challenging activity	1	Can be scored on a 6–20 category scale or a 0–10 category-ratio scale	Raw score. Higher score indicates greater exertion, fatigue, or breathlessness	Right now	1 publication, used as a fatigue scale [55]
Chronic Respiratory Questionnaire [56, 57]	Interviewer-led or PROM	Adults	Dyspnea, fatigue, emotional function, mastery (feeling of control over disease)	20	Yes/No, 7-point scale, includes an open-ended question and a section that permits respondents to select 5 activities from a list of 25 and rate breathlessness during that activity	Total score and domain scores, Higher scores indicate better HRQOL	2 weeks	2 publications [58, 59]
COPD Assessment Test [60]	PROM	Adults with COPD	Cough, phlegm, chest tightness, breathlessness going up hills/stairs, activity limitation at home, confidence leaving home, sleep, and energy	8	0–5 rating of status	Total score. Higher scores indicate greater disease severity	None	21 publications Key studies include: [13, 24, 61–63]
Clinical COPD Questionnaire [64]	PROM	Adults with COPD	Symptoms, functional state, and mental state	10	0–7 Likert-type for frequency	Total average score, average domain scores. Higher scores indicate worse HRQOL	1 week	1 publication [59]
COPD Severity Score [65–67]	PROM	Adults with COPD	Severity of respiratory symptoms, systemic steroid use, other medication use, prior hospitalization or intubation, home oxygen use	5	Varies by question	Score of 0–35, items are weighted. Higher scores indicate greater COPD severity	2 weeks to 5 years, by question	7 publications Key studies include: [24, 68, 69]
Epworth Sleepiness Scale [70]	PROM	Adults	Situations in which people may fall asleep	8	0–3 Likert-type for chance of falling asleep	Total score 0–24. Higher score indicates greater sleepiness	None	2 publications [53, 71]
EQ-5D-5L [72]	PROM	Adults	Mobility, self-care, ability to do usual activities, pain/discomfort, anxiety/depression, and overall health	25; shorter versions are available	5-point scale	Sum score converted to an index utility score. Higher scores indicate better quality of life	None	5 publications Key studies include: [24, 73, 74]
Hamilton Anxiety Rating Scale [75]	ClinRO, has been used as a PROM	Any	Anxiety symptoms	14	0–4 rating of severity	Total score 0–56. Higher scores indicate more severe anxiety	None	1 publication [34]
Hamilton Depression Rating Scale [76]	ClinRO	Adults	Signs and symptoms of depression	17, a 21-item version is also avail-able	Varies by question	Total score. Higher score indicates greater severity of depression	None	1 publication [34]
Hospital Anxiety and Depression Scale [77]	PROM	Adults	Anxiety, depression	14	0–3 rating of impairment	Total score, subscale scores. Higher score indicates greater impairment	None, 1 week has been used	2 publications [44, 78]
Leicester Cough Questionnaire [79]	PROM	People with chronic cough	Frequency of cough symptoms and impacts in physical, psychological, and social domains	19	7-point Likert-type for frequency	Total score range of 3–21. Higher score indicates lesser impairment	2 weeks	1 publication [80]
Living with COPD Questionnaire [81]	PROM	People with COPD	Ability to meet daily needs for self-actualization, safety/security, independence, self-esteem, control, and social/relationship	22	True, Not True	Total score 0–22. Higher score indicates greater impact	“At the moment”	2 publications [14, 24]
Memorial Symptom Assessment Scale; Revised Memorial Symptom Assessment Scale [82, 83]	PROM	People with cancer; RMSAS for people with COPD	Frequency and distress of physical and psychological symptoms	32; a 24 item short form is also available	Yes/No for occurrence; 1–4 Likert-type for frequency, 1–4 Likert-type for severity, 0–4 Likert-type for bothersome-ness	Total score; Subscales: global distress index (composite of 4 psychological and 6 physical symptoms); domain scores for physical and psychological symptoms	1 week	1 publication [84]
Modified Medical Research Council dyspnea scale [85]	Single item rating scale	Any health condition	Dyspnea during activity; the modified version uses plain language	1	Patient selects from 5 statements describing activity levels	0–4 item score. Higher score indicates greater impairment of activity	None	15 publications Key studies include [26, 45, 61]:
Mishel Uncertainty of Illness Scale [86]	PROM	Adults, adolescents	Ambiguity, complexity, inconsistency, and unpredictability	33, 32, 30, 28, and 5-item versions	5-point Likert-type for agreement	Total score. Higher scores indicate greater uncertainty	Today	3 publications [44, 87, 88]
Pittsburgh Sleep Quality Index [89]	PROM	Adults	Sleep quality, sleep latency, sleep duration, habitual sleep efficiency, sleep disturbances, use of medication, daytime dysfunction	19 scored items	Varies by question, includes open-ended responses	0–21 summary score. Higher scores indicate worse sleep	1 month	1 publication [53]
Quality of Life—Bronchiectasis v.3.1 [90, 91]	PROM	People with bronchiectasis	Respiratory symptoms, physical functioning, vitality, role functioning, emotional functioning, social functioning, health perceptions, treatment burden	36	Varies by question	Items scored 1–4; scale scores converted to 0–100 scale. Higher scores indicate better HRQOL	1 week	1 publication [80]
SF-36 [92]	PROM	Adults	Vitality, bodily pain, general health, mental health, physical functioning, physical role functioning, emotional role functioning, social role functioning	36	Yes/no and Likert-type	Summary score; domain scores; algorithm conversion to 100-point score (v2). Higher scores indicate better health status	1 week, 4 weeks	5 publications found for three studies [26, 38, 93–95]
St. George’s Respiratory Questionnaire [96]	PROM	Adults with respiratory diseases	Respiratory symptoms, activities associated with dyspnea, and psychosocial impacts of disease	50 items plus a health status question; a 40-item short form is available and has been validated for COPD	Varies by question	0–100. Higher score indicates greater limitations	None	49 publications Key recent studies include: [15, 35, 97, 98]

^a^2010–2023

**Table 4:** Clinical trials of treatment for AATD that included a PROM

NCT/EUDRA number	Title	Phase	PROs/COAs used	PRO endpoint(s)	Assessment timing	Endpoint position(s)
NCT02796937	Long Term Safety of Alpha1-Proteinase Inhibitor in Subjects With Alpha1 Antitrypsin Deficiency	Phase 3	St. George's Respiratory Questionnaire (SGRQ-C); EQ-5D-5L; tentative: number and severity of COPD exacerbations (manner of data collection not specified)	Change from baseline for SGRQ and EQ-5D-5L	Baseline and Week 52 and 104; exacerbations collected week 2 through week 108	Secondary
NCT03636347	A 12-week Study Treating Participants Who Have alpha1-antitrypsin-related COPD With Alvelestat (MPH966) or Placebo	Phase 2	St. George's Respiratory Questionnaire (SGRQ-C)	Change from baseline in St. George’s Respiratory Questionnaire (SGRQ-C) to end of treatment (12 weeks)	Baseline, 12 weeks	Exploratory
NCT03114020	Efficacy/Safety of HA Inhalation Solution for Hereditary Emphysema in Patients With Alpha-1 Antitrypsin Deficiency	Phase 2	St. George's Respiratory Questionnaire	Not specified	28 days	Secondary
NCT01983241	Efficacy and Safety of Alpha1-Proteinase Inhibitor (Human), Modified Process (Alpha-1 MP) in Subjects With Pulmonary Emphysema Due to Alpha1 Antitrypsin Deficiency (AATD)	Phase 3	St. George's Respiratory Questionnaire (SGRQ); EQ-5D-5L	Change from baseline in SGRQ; change from baseline in EQ-5D-5L	Baseline, Weeks 26, 52, 78, 104, 130 and 156	Secondary
EUDRA 2018-001309-95	A Phase 2, proof-of-concept, multicentre, double-blind, randomised, dose-ascending, sequential group, placebo-controlled study to evaluate the mechanistic effect, safety, and tolerability of 12 weeks twice daily oral administration of alvelestat (MPH966) in participants with alpha-1 antitrypsin deficiency	Phase 2	St. George's Respiratory Questionnaire (SGRQ-C)	Change from baseline in St. George’s Respiratory Questionnaire (SGRQ-C) at end of treatment	Baseline, end of treatment	Secondary

Trials commencing 2010 to March 2023

## Discussion

This scoping review identified 44 concepts that reflect symptoms and impacts of AATD and AATD-related lung disease, and provide a broad picture of life with this health condition. These concepts demonstrate that a diagnosis of AATD conveys burdens for patients, including the genetic diagnosis itself and treatment burden, and that AATD-related lung disease results in additional symptoms and impacts related to lung function. Our findings also indicate that some symptoms and impacts of lung disease may differ between people with and without AATD. These differences appear linked to the age of disease onset, disease severity and rate of progression.

These general findings highlight limitations of the literature, specifically the lack of published, in-depth qualitative studies examining the lived experiences of people with AATD. This poses a significant obstacle to understanding which outcomes are most meaningful to patients in relation to treatment benefit, a necessary precursor to selecting a PROM for use in a clinical trial. For example, qualitative study findings suggest that anxiety experiences associated with a genetic diagnosis are important, but it is unclear whether people with AATD regard these as relevant to the assessment of treatment outcomes. Similarly, AATD-related lung disease progression may be affected by exacerbation frequency, but it is unclear how exacerbations should be assessed or whether patients themselves are able to report them reliably. Choate et al. [27] reported that people with AATD and lung impairment recognized about half of the exacerbations they experienced, with low correlation between health care utilization based and symptoms-based definitions of exacerbation.

Although several PROMs have been used in studies of people with AATD and lung disease, these PROMs are not specific to AATD, and the review identified only two PROMs that had been used in clinical trials of AATD treatment (i.e., EQ5D-5L and SGRQ). Qualitative and psychometric studies of PROMs within the AATD population appear to be lacking, and are needed to understand the relevance of measure content and the meaningfulness of changes in score for this population. Finally, from the patient’s perspective, an ideal treatment pathway for AATD might include early identification of the genetic disorder followed by treatment that effectively prevents disease altogether, greatly extends the period of disease-free years, or greatly slows the progression of early lung symptoms and impacts. For such a treatment, currently available PROMs may not be applicable.

### Limitations of the study

Because of the limited number of published qualitative studies of people with AATD, evidence from studies that used questionnaires to gather data as well as other types of resources, such as the AlphaNet patient guide, were used to identify potentially relevant outcome concepts. These sources of information do not provide ‘gold standard’ evidence for the outcomes that matter to patients because they are subject to various forms of limitation and bias. For example, questionnaire-based studies limit the outcomes that people can report to those selected by researchers and non-traditional resources may blend the perspectives of clinicians or other stakeholders with those of patients. Additional qualitative studies are needed to affirm or expand the understanding of the experiences of people with AATD, including explorations of how they experience COPD and what treatment outcomes are most important to them.

This study focused on reviewing literature related to experiences of AATD and AATD-related lung disease. Literature focused on the experiences of people with other AATD-related illnesses may have provided additional relevant insights on selected topics, such as treatment burden associated with AAT augmentation therapy, and may have resulted in the identification of additional measures.

## Conclusions

People with AATD experience a variety of burdens associated with the genetic diagnosis itself and with lung impairments or disease that may develop due to AATD. However, the limited amount and scope of qualitative study publications focused on patient experiences of AATD and associated lung disease hampers understanding of how best to assess patient-centered outcomes of treatment among populations of people with AATD. No AATD disease-specific PROMs were identified. A limited number of PROMs have been used in research on AATD, including COPD-specific measures that assess symptoms and quality of life and measures of mood, sleep, and general physical and psychosocial functioning. Most of these PROMs have been used in only one or two studies. In clinical trials of AATD treatments, the use of PROMs was limited, with just two PROMs being used over more than a decade. As a result, there is little information to guide investigators who wish to select a PROM for use in understanding the experiences of people with AATD or for evaluating treatment benefits for this population.

To address these gaps, well-designed qualitative studies are needed to elicit comprehensive data about the lived experiences and treatment priorities of diverse samples of people with AATD, both with and without lung disease. Incorporating patients’ lived experience in drug development is a key goal of the Critical Path for Alpha-1 Antitrypsin Deficiency (CPA-1) Consortium and part of FDA patient focused drug development guidance [49, 50]. Such studies would provide crucial data needed to understand the outcomes that matter to people with AATD and to evaluate the alignment between these outcomes and the available PROMs. For any PROMs that appear to have suitable content, additional qualitative or psychometric studies may also be needed to evaluate their measurement properties. Finally, a better understanding of the experiences of people with AATD, assessed both qualitatively and quantitatively, could help identify concepts of interest that are relevant to different pathways for treatment or identify potential new pathways for treatment, including non-pharmacological therapies and improvements to healthcare or treatment delivery, such as making it easier for people to obtain augmentation therapy at home.

## Supplementary Information

Additional file1 (DOCX 49 kb)

## Data Availability

All data extracted from the included studies are available via PubMed, Embase, Google Scholar, or websites as listed in the references section.

## Abbreviations

AAT: Alpha-1 antitrypsin
AATD: Alpha-1 antitrypsin deficiency
CAT: COPD assessment test
COPD: Chronic obstructive pulmonary disease
FDA: U.S. Good and Drug Administration
HRQOL: Health related quality of life
PROM: Patient reported outcome measure
SGRQ: St. George’s Respiratory Questionnaire
SGRQ-C: St. George’s Respiratory Questionnaire, COPD version
