# Supplementary material for: Patient-centered assessment of treatment for alpha-1 antitrypsin deficiency: literature review to identify concepts and measures for people with alpha1-antitrypsin deficiency
Source: Orphanet J Rare Dis. 2025 Feb 22;20:83. doi: 10.1186/s13023-025-03592-9 (PMC11846308; doi:10.1186/s13023-025-03592-9)
Supplement: Supplementary file 1 — Additional file1 (DOCX 49 kb) [file 13023_2025_3592_MOESM1_ESM.docx]

# Supplementary File Content

Supplementary File 1

Table 1. Search Concepts, Terms, and Operators for AATD with Lung Disease

| Search Steps | Terms and operators |
| --- | --- |
| 1. | “alpha-1 antitrypsin deficiency” OR AATD OR AAT OR "alpha 1 antitrypsin deficiency" |
| 2. | (Lung AND (disease OR function OR impairment OR inflammation OR damage)) OR emphysema OR COPD OR “chronic obstructive pulmonary disease” OR asthma OR “chronic bronchitis” OR bronchiectasis |
| 3. | 1 AND 2 AND (filters: English, 2010 to date of search) |

Table 2. Search Strategies to Identify Qualitative & HRQOL Articles in the Resource Database

| Topic | Terms and operators |
| --- | --- |
| Quality of life | “quality of life” OR HRQOL or QOL or “well-being” or “health status” |
| Patient experiences | “patient experience” OR “patient perspective” |
| Qualitative study | qualitative OR interview OR “focus group” OR “patient engagement” |
| Disability / Function | disability OR functioning OR “activities of daily living” or mobility |
| Symptom burden | “symptom burden” OR “illness burden” OR “disease burden” OR “symptom impact” OR “disease impact” |

HRQOL – health related quality of life

Table 3. Overview of Included Publications

| **Short citation** | **Study Design** | **Data Source** | **Concepts found or reported** | **Measures used (if any)** |
| --- | --- | --- | --- | --- |
| Anzueto 2015 | Review | Literature | Difficulty with activities of daily living, difficulty raising children, needing assistance/caregiver, missed workdays due to treatment, treatment burden on time, costs, impact on family/caregivers, dyspnea, wheezing, cough, volume of sputum |  |
| Barjaktarevic 2021 | Review | Literature | Impact on work/career choice, avoidance of environmental exposures, exercise, number of exacerbations, healthcare utilization, use of supplementary oxygen, fatigue, mood, sense of control over condition, dyspnea, weight loss, malnutrition, sputum |  |
| Gøtzsche 2010 | Review | Literature | Number of exacerbations, SGRQ score | St. George's Respiratory Questionnaire |
| Gøtzsche 2016 | Review | Literature | Number of exacerbations, SGRQ score | St. George's Respiratory Questionnaire |
| Holm 2013 | Observational cross-sectional | Self-report questionnaires | Depression, anxiety, use of supplemental oxygen, dyspnea, quality of life | Modified Medical Research Council Dyspnea Scale; St. George's Respiratory Questionnaire; Hospital Anxiety and Depression Scale |
| Manca 2014 | Observational cross-sectional | Measures administered during face to face interviews | Predictors of PROM scores were FEV1(%) and pack-years of smoking; patients with AATD exhibited correlations between symptomatic impairments and quality of life scores | COPD Severity Score; EQ-5D; Living with COPD (LCOPD); COPD Assessment Test; Modified Medical Research Council Dyspnea Scale; Charlson Co-morbidity Index |
| Tian 2020 | Observational cross-sectional | Survey | Perceived stress, life satisfaction, stigma/genetic discrimination, social support resources, marital quality | Questions were selected or adapted from a variety of in other publications to create study-specific assessments |
| Torres Redondo 2017 | Observational cross-sectional | Self-report questionnaires | Quality of life/physical functioning, dyspnea | SF-36; Modified Medical Research Council Dyspnea Scale |
| Choate 2021 | Longitudinal cohort study | Monthly data collection conducted via telephone | Number of exacerbations, dyspnea, cough; 48% of events reported over the 1-year study were described by participants as exacerbations and 68% of participants experienced at least 1 exacerbation during the year |  |
| Choate 2022 | Longitudinal cohort study | Monthly data collection | Use of supplemental oxygen, use of inhaled medication, number of exacerbations, dyspnea, increased sputum, change in sputum color |  |
| Stoller 1994 | Observational cross-sectional | Survey by mail sent to Alpha-1 National Association newsletter subscribers | Job impact (retired early, lost job, changed to an easier job), impact on health insurance, impact on social life/friendships (lost friend, made a friend), impact on marital/family life (worse marriage/better marriage), dyspnea, wheezing all the time, wheezing with colds, cough, phlegm, |  |
| Raveendran 2023 | Qualitative | Interviews | Focused on concepts unrelated to disease experience or treatment efficacy, including barriers to diagnosis, treatment history, satisfaction with health care, and willingness to share data with a patient registry |  |
| Wienke 2014 | Qualitative | Interviews and focus groups | Stigma, guilt, fear for future generations, fear for children's health, strain within the family dynamic, impacts on patient/family relationship, avoidance of environmental exposures, vigilance, behavior modifications related to susceptibility, feeling fatalistic, symptoms (not specified), cough, sadness/depression, anger, uncertainty, "redemptive adjustment" (purpose and meaning to life created by having social challenges that result from AATD) |  |
| Herepath 2020 | Review | Literature | No specific concepts reported in this conference abstract, broad categories only (e.g., complications) |  |
| Worthington 2018 | Observational cross-sectional | Survey | Unpredictability, subjective numeracy, math anxiety, spiritual well-being, negative affect (afraid, worried, fearful), affect-management coping (wanting to feel hopeful, desiring peace of mind) |  |
| Buchman 2013 | Observational cross-sectional | Measures administered during phone interviews | Depression, anxiety; 45% of participants experienced depression and most of them reported mild depression, 15% of participants experienced moderate anxiety | Hamilton Depression Rating Scale (HAM-D), Hamilton Anxiety Rating Scale (HAM-A) |
| Sandhaus 2020a | Longitudinal cohort study | Registry data collected monthly by phone | Quality of life assessed by SGRQ; analysis indicated that annual worsening of score was larger in the control group compared with group receiving AAT augmentation therapy | St. George's Respiratory Questionnaire |
| Sandhaus 2020b | Review | NA | Number of exacerbations, duration of exacerbations, burden of treatment (travel and time for infusion therapy, convenience of home therapy administration), ability to work, ability to perform family roles, well-being/quality of life, ability to travel, ability to feel independent/control life, satisfaction with treatment, exercise, ability to carry out daily activities, muscle weakness, anxiety, depression, dyspnea, new/increased sputum, change in sputum color |  |
| Beiko 2019a | Observational cross-sectional | Surveys administered by a confidential testing study | Anxiety; higher anxiety scores were significantly correlated with female sex, lower income, and smoker/past smoker status; no correlation with genotype or age. Anxiety scores were significantly correlated with SF-12 mental health composite scale scores, but not physical health composite scale scores | Beck Anxiety Inventory, SF-12 |
| Redondo 2015 | Observational cross-sectional | Self-report questionnaires | Study found sex-specific differences in SF-36 domain scores for bodily pain, vitality, and mental health; there were differences for ZZ patients with and without augmentation therapy on change in health; FEV1(%) was significantly and positively correlated with physical functioning, role physical, and general health; number of hospitalizations in the previous year also influenced SF-36 scores | SF-36 |
| Molloy 2017 | Observational cross-sectional | Survey | Symptoms (not specified), ability to work; also reported as important to patients: slower progression of disease, fewer chest infections, reduced number of hospitalizations |  |
| Sandhaus 2022 | Qualitative and non-randomized experimental | Outcome assessments and qualitative data | Nasal dryness/moisture, mouth dryness/moisture, comfort using the device (a humidifier for nasal oxygen administration) at night, difficulty using the device during activity, improved sleep, waking without a dry nose |  |
| Beiko 2019b | Review | Literature | Fatigue, anxiety, depression, ambiguity/uncertainty, altered mood, quality of life, impacts on daily life and role functioning including ability to work, burden of treatment, avoidance of environmental exposures, impact on family/lifestyle choices, symptom burden, number of exacerbations, dyspnea |  |
| Gauvain 2015 | Observational cross-sectional | Surveys | Number of exacerbations, ability to walk, quality of life assessed by SGRQ, dyspnea, wheezing | St. George's Respiratory Questionnaire |
| Alpha-1 Foundation 2023 | NA | NA | Stress, depression, anxiety, fear, lifestyle or job changes, exercise, impact on family/family relationships, managing risks/environmental exposures, family planning/pregnancy decisions, number of exacerbations, fatigue/level of energy, stamina/need to rest during normal activity, use of acute treatments, burden of treatment, use of supplemental oxygen, use of airway clearance device/method, muscle weakness, ability to concentrate, memory, mood, ability to carry out daily activities or work tasks, sleep quality, sleep disturbance, ability to maintain good nutritional status, difficulty with mobility, panic attacks, needing assistance, anger/frustration, libido and sexual function, ability to travel, dyspnea, tightness in the chest, wheezing, sputum, color of sputum, cough, irritability/change in personality, fluid retention/edema, cognitive changes (forgetfulness, confusion, sleepiness, slurred speech), fever, respiratory rate, cyanosis, headache, dizziness, restlessness, rapid heart rate, congestion, unintentional weight loss |  |
| O'Connor 2011 | Observational cross-sectional | Self-report questionnaires | Study showed that asymptomatic people with AATD had higher quality of life as measured by SGRQ than symptomatic people | St. George's Respiratory Questionnaire |
| Hoth 2013 | Observational cross-sectional | Surveys | Illness uncertainty, complexity, ambiguity, depression, anxiety, quality of life; results indicated that ambiguity/uncertainty about physical symptoms was a significant predictor of other outcomes, but complexity was not a predictor of any outcome, dyspnea | Mishel Uncertainty in Illness Scale; St. George's Respiratory Questionnaire; Modified Medical Research Council Dyspnea Scale; Hospital Anxiety and Depression Scale |
| Mobeen 2021 | Observational cross-sectional | Registry data | Depression, anxiety, dyspnea |  |
| Williams 2013 | Qualitative | Photovoice, focus groups | Family impact from disclosure, genetic condition-related decision-making burden, family planning/pregnancy decision making, social impact from perceived susceptibility to environmental exposures, stigma, adaptation to condition, behavioral changes; guilt, family conflict/strain, avoidance of environmental risks, ability to have/keep pets, treatment burdens (e.g., from supplemental oxygen equipment) |  |
| FDA 2016 | NA | NA | Exercise, cough, dyspnea, number of exacerbations, exhaustion (fatigue/level of energy), anxiety, costs, convenience of therapy administration, side effects, treatment burden on time, long acting treatment, fear, frustration, stress, difficulty raising children, daily life and role functioning, avoidance of environmental exposures, vigilance, ability to carry out daily activities, ability to travel, ability to work, anger/frustration, fear, impact on family/caregivers, libido and sexual function, sputum, stress, burden of treatment, family planning/pregnancy decisions, depression, ability to feel independent, ability to walk, exertion (stairs, carrying heavy things), lifestyle or job changes, dyspnea |  |
